# Assessing the Role of the ‘Unity Assumption’ on Multisensory Integration: A Review

**DOI:** 10.3389/fpsyg.2017.00445

**Published:** 2017-03-31

**Authors:** Yi-Chuan Chen, Charles Spence

**Affiliations:** Crossmodal Research Laboratory, Department of Experimental Psychology, Oxford UniversityOxford, UK

**Keywords:** the unity effect, the unity assumption, coupling priors, crossmodal correspondences, semantic congruency

## Abstract

There has been longstanding interest from both experimental psychologists and cognitive neuroscientists in the potential modulatory role of various top–down factors on multisensory integration/perception in humans. One such top–down influence, often referred to in the literature as the ‘unity assumption,’ is thought to occur in those situations in which an observer considers that various of the unisensory stimuli that they have been presented with belong to one and the same object or event (Welch and Warren, 1980). Here, we review the possible factors that may lead to the emergence of the unity assumption. We then critically evaluate the evidence concerning the consequences of the unity assumption from studies of the spatial and temporal ventriloquism effects, from the McGurk effect, and from the Colavita visual dominance paradigm. The research that has been published to date using these tasks provides support for the claim that the unity assumption influences multisensory perception under at least a subset of experimental conditions. We then consider whether the notion has been superseded in recent years by the introduction of priors in Bayesian causal inference models of human multisensory perception. We suggest that the prior of common cause (that is, the prior concerning whether multisensory signals originate from the same source or not) offers the most useful way to quantify the unity assumption as a continuous cognitive variable.

## Introduction

The ‘unity assumption’ is an observer’s assumption, or belief, that two or more unisensory cues belong together (i.e., that they come from the same object or event^1^, Welch and Warren, 1980, 1986; Spence, 2007; Chen and Vroomen, 2013). Such an assumption, or belief^2^, on the part of the observer serves as a cognitive modulator of multisensory integration, leading to the empirical observations described as the ‘unity effect’. The unity assumption certainly serves as one of the key mechanisms by which the human brain solves the crossmodal binding problem; that is, how signals from the different senses are encoded into a unified object/event representation (Senkowski et al., 2008; Spence, 2011). Furthermore, the unity assumption provides a good example pertinent to the long-standing debate concerning the role of cognitive penetration on human perception. To date, though, visual rather than multisensory cases have constituted the primary focus in previous reviews (e.g., Bruner, 1957; Fodor, 1983; Pylyshyn, 1999; Vetter and Newen, 2014; Firestone and Scholl, 2016).

**FIGURE 1:**
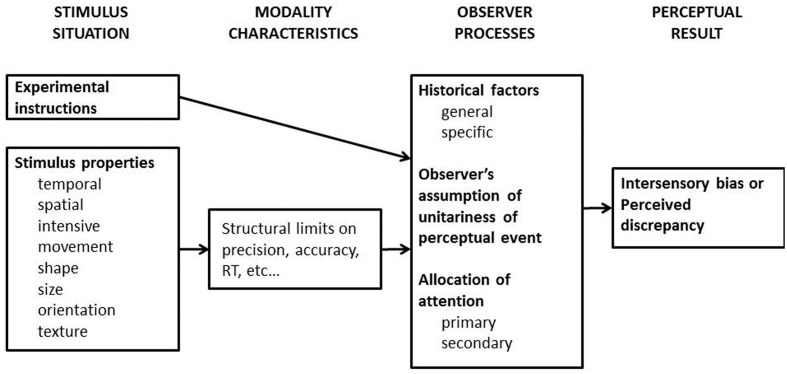
**Welch and Warren’s (1980) early model of multisensory interactions concerning those situations in which “intersensory bias would occur.”** The first stage which pertains to *stimulus situation* includes the descriptive characteristics of the signals that will be received by multiple sensory systems (i.e., these are so-called ‘amodal’ features), and the observer’s current goal. Notice here that spatial and temporal coincidences were listed at this first stage. This constrains what goes on at later stages of information processing. The second stage, *modality characteristics*, determines how the sensory signals are received and represented, such as that the shape of a 3-D object is perceived as a 2-D visual array initially by the visual system, but its surface and edge are perceived by the cutaneous and proprioceptive systems. The third stage, *observer processes*, concerns how human brains process/integrate the information from different modalities in order to fit the task goal. The *general* historical factors refer to the long-term likelihood that the information from different sensory modalities should go together; by contrast, the *specific* historical factors refer to the observer’s past experience regarding a particular stimulus pair, such as one’s pet dog and its unique barks, should undoubtedly go together. The model also suggests that the observer’s attention is *primarily* allocated to the modality that is typically most appropriate to the current task, such as vision in spatial tasks and audition in temporal tasks. Nevertheless, experimenter’s instruction or task demands may leads to the shift of attention to another sensory modality (i.e., *secondary* attention). The *unity assumption* factor listed at this stage is the main interest of the current review paper. These serial processes lead to a perceptual outcome that either the discrepant information from different sensory modalities is integrated, so intersensory bias would be observed, or, instead, the discrepant information is represented separately, so the discrepancy between the two sensory stimuli would be detected. Back in the 1980s, feed-forward models were the predominant view given the popular and rapidly developing approach of computational neural models. Nowadays, of course, we realize that feedback may be just as, if not even more, important (e.g., Talsma et al., 2010). This figure is reproduced from Figure 1 in Welch and Warren (1980).

More generally, the unity assumption can be thought of as one of a number of factors that influence the binding of multisensory cues (see **Figure 1**; see also Spence, 2007, for a review). Over the last 30 years or so, researchers have generally tended to focus their attention on the role of spatiotemporal coincidence on multisensory integration (see Stein and Meredith, 1993; Calvert et al., 2004; Bremner et al., 2012; Stein, 2012, for reviews). Nevertheless, the last 10 years has seen a rapid growth of interest in the role of various higher-level factors, such as semantic congruency (e.g., a dog and a barking sound, Doehrmann and Naumer, 2008; Chen and Spence, 2010, Chen and Spence, 2011b; Naumer and Kaiser, 2010), crossmodal correspondences (e.g., based on the internalization of the statistical regularity between pitch and size, Spence, 2011; Parise and Spence, 2013), and the ‘unity assumption’ (e.g., Vatakis and Spence, 2007) in multisensory integration. Admittedly, it can sometimes be difficult to clearly distinguish between the latter factors^3^. Here, we will critically assess whether research on the topics of crossmodal correspondences and semantic congruency should also be considered as relevant to the debate concerning the role of the unity assumption in multisensory integration.

A growing body of empirical research, utilizing a wide range of different experimental paradigms, demonstrates that the unity assumption modulates multisensory integration under at least a subset of experimental conditions. In this review, we critically evaluate the sometimes conflicting evidence from studies of the ventriloquism effect (both spatial and temporal; Jackson, 1953; Morein-Zamir et al., 2003), the McGurk effect (McGurk and MacDonald, 1976), and the Colavita visual dominance effect (e.g., Colavita, 1974; Spence et al., 2011). These empirical results help answer the question of the conditions under which the unity assumption is formed and modulates multisensory integration in humans.

In recent years, researchers have suggested that assumptions concerning the multisensory inputs that likely belong together could be represented as ‘coupling priors’ or ‘prior of common cause’ according to Bayesian models (e.g., Ernst, 2007; Sato et al., 2007; Shams and Beierholm, 2010). The Bayesian approach certainly provides one means of formalizing different degrees of certainty regarding the unity of two or more unisensory inputs as a continuous (rather than as a discrete) variable (e.g., Körding et al., 2007). It should, however, be noted that the Bayesian approach is not without its critics. Some, for instance, have argued that Bayesian models simply re-express the cognitive model in mathematical language (see Murphy, 1993; Jones and Love, 2011; Bowers and Davis, 2012).

Taken together, there is, then, currently good evidence to support the view that the unity assumption influences multisensory integration under at least a subset of experimental conditions. Here, we address the question of why null results have sometimes been reported in previous studies. We also raise the question of whether the notion of the ‘unity assumption’ still has value in the era of ‘priors’ in Bayesian models. We question whether these terms are, in fact, co-terminous. We also evaluate the evidence concerning how rapidly the unity effect can be demonstrated when formerly unrelated pairs of sensory stimuli are experienced together (that is, when they are presented together). Finally, we highlight some of the key questions awaiting future research in this area.

## Factors Leading to the Unity Assumption

It has long been argued that whenever two or more sensory inputs are considered as in some sense ‘going together,’ observers will be more likely to treat them as referring to a single multisensory object or event, as compared to the condition where such an assumption is lacking (e.g., Thomas, 1941; Jackson, 1953; Welch and Warren, 1980, for early examples). Consequently, an observer will be more likely to infer that the sensory inputs have a common spatiotemporal origin as well. Hence, they will be more likely to bind the inputs into a single multisensory object/event representation (see Bedford, 2001).

The situation at the cinema, where we hear the voices as coming from the lips of the actors that we see talking on the screen (rather than from the loudspeakers situated elsewhere in the auditorium, known as the *spatial ventriloquism* effect), provides an illustrative everyday example here. Many commentators have taken this common experience as evidence of the unity effect in action. They often point to the apparent visual capture of auditory localization that is commonly experienced in such situations. However, it is worth noting that this may be more of an unconsidered assumption than an empirically demonstrated phenomenon. Indeed, back in the psychophysics laboratory, one tends to see partial capture, with the sound being moved just a few degrees toward the perceived location of the simultaneously presented visual stimulus (e.g., see Jackson, 1953; Bertelson and Aschersleben, 1998; Alais and Burr, 2004). Some studies have also reported that the visual stimulus is mislocated slightly toward the auditory stimulus as well (e.g., Bertelson and Radeau, 1981).

In early studies, the unity effect was mainly demonstrated by experimenters who would provide their participants with *explicit* instructions that the sensory inputs from different modalities either came from the same or from different sources (see Welch and Warren, 1980, for an early review). Such experimenter-induced beliefs were, for example, reported to affect the amount of adaptation that was seen following exposure to both audiovisual (Radeau and Bertelson, 1974) and visual-proprioceptive conflict (Welch, 1972). On the other hand, the unity effect can also be induced *implicitly* by stimuli with highly congruent properties, such as their redundancy in terms of temporal synchrony, and the observer’s prior experiences (either long-term knowledge-based or short-term contextual-based).

In the knowledge-based cases, for example, crossmodal correspondences and semantic congruency are two of the factors that plausibly give rise to different levels of congruency concerning the crossmodal sensory inputs. Such a unity assumption induced by the properties of the stimuli was also proposed in Welch and Warren’s seminal review paper, where the researchers talked of the “*compellingness of the stimulus situation.*” They went on to suggest that “*a highly compelling situation is one in which the assumption of unity is strong*” (Welch and Warren, 1980, p. 649; see also Warren et al., 1981). As in the above example, the video and audio presented in a cinema constitutes a highly compelling situation, since there seems no better assumption regarding the source of the voice other than that it came from the actor’s lips. Unfortunately, though, Welch and Warren provided no independent means of characterizing the compellingness of a particular pairing of unisensory stimuli, thus meaning that the term is pretty much useless (or, rather, unconstrained), practically speaking.

### Experimenter Instructions

Welch and Warren (1980) reviewed those early studies in which the participant’s belief regarding the common or separate sources of the multisensory inputs was manipulated explicitly, and different perceptual outcomes were demonstrated. For example, Miller (1972) had the participants in his study see and feel different shapes. When the participants were instructed that “*they would be seeing and feeling identical halves of the same object*” (p. 121), their performance on the shape matching task demonstrated visual dominance (e.g., Rock and Victor, 1964). However, when the participants were instructed ambiguously “*to look at ‘something’ and feel ‘something’ and then to match ‘the object”’* (p. 122), they were able to accurately report on the shape that they were feeling (i.e., with no bias by the visual information). Note that the use of instructions in order to try and encourage participants to integrate multisensory information is still sometimes used, as in those studies that have wanted to test optimal statistical integration based on Bayesian models (e.g., Alais and Burr, 2004, to ask their participants to “*think the display as a ball thudding onto the screen*,” p. 260).

Miller’s (1972) results provide a powerful demonstration of the modulatory role of the experimenter’s instructions on multisensory integration. However, in Warren et al.’s (1981) study, the instructions provided by the experimenter were shown to modulate the spatial ventriloquism effect only in certain conditions when multisensory speech stimuli were used. Specifically, they demonstrated a larger spatial bias of auditory localization by vision when the participants were instructed that the stimuli came from the same event rather than separate events (see the section “The Spatial Ventriloquism Effect”). Nevertheless, the instructions given by the experimenter modulated spatial ventriloquism only when the video of a speaker’s face and voice were presented synchronously rather than asynchronously, and only when the speaker’s face was presented rather than when it was replaced by a piece of tape. In summary, instructions concerning whether multisensory signals belong to the same object/event or not constitute an explicit and exceedingly simple means by which to demonstrate the unity effect. Importantly, however, it may not be sufficient (e.g., Warren et al., 1981).

### Redundant Information

Inputs from different sensory modalities sometimes provide information about the same attribute or feature, thus potentially giving rise to informational redundancy. Crossmodal redundancy occurs primarily in those domains that some researchers like to call *amodal*, such as space and time, as well as stimulus intensity, size, and shape (see Walker-Andrews, 1994; Spence, 2011). Spatial and temporal coincidence, for example, are two well-recognized factors that can enhance multisensory integration (see Stein and Meredith, 1993; Spence, 2007, for reviews; though see also Spence, 2013). In the model proposed by Welch and Warren (1980, see **Figure 1**), spatial and temporal coincidence are listed under those factors that belong to the stimulus situation. Specifically, multisensory stimuli that are presented close in time or space may have been encoded and/or integrated during the feed-forward processing (i.e., in a bottom–up fashion, see Stein and Meredith, 1993; Noesselt et al., 2010; van Atteveldt et al., 2014). Nevertheless, it is important to note that spatial and temporal coincidence have also been designated as cognitive factors. For example, in Warren et al.’s (1981) study, the visual and auditory stimuli were either presented synchronously or else asynchronously (with a 150 ms delay in one of the signals). The suggestion was that the former condition would deliver a higher degree of *compellingness* (leading to a stronger assumption of unity) than the latter situation.

More recent studies have provided evidence that the unity assumption regarding visual and tactile signals is stronger when the participants view their own hand grasping or exploring an object. So, for example, Helbig and Ernst (2007) demonstrated that the unity assumption induced by such means powerfully modulated visual-tactile integration, irrespective of whether the perceived location of the visual and tactile object was the same or different (i.e., mirror reflection could be used to make the visual object(s) appear at an illusory location). Subsequent studies demonstrated that the forming of the unity assumption depends on the participants seeing their own exploratory hand movements (i.e., the congruent visual and proprioceptive information) rather than their “knowing” that what is seen and touched necessarily refer to the same object (cf. Miller, 1972; Misceo and Taylor, 2011; Misceo et al., 2014). Combined, seeing and feeling the object via exploratory movement at the same time appears critical to inducing a unity effect for visuotactile integration (see Lacey and Sathian, 2014, for a review).

### Crossmodal Correspondences

**FIGURE 2:**
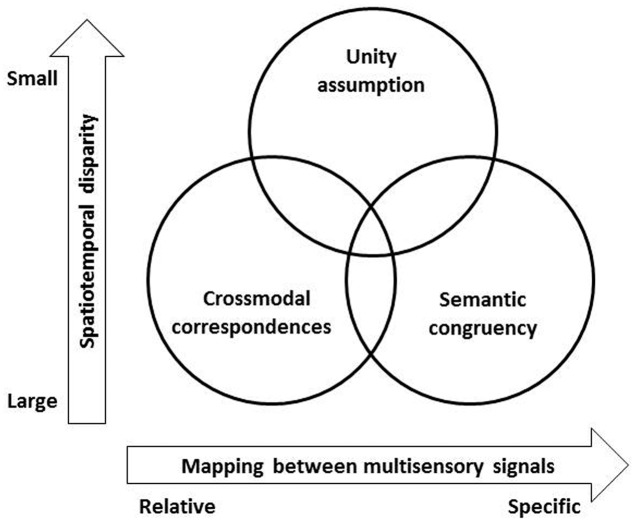
**A schematic figure using two dimensions to represent the relationships between the three top–down modulatory factors in studies of multisensory perception: the unity assumption, crossmodal correspondences, and semantic congruency.** The X-axis highlights the fact that crossmodal correspondences typically constitute relative, rather than absolute, mappings between modality-specific dimensions (such as higher pitch and larger size, see Gallace and Spence, 2006), while semantically congruent stimuli refer to those features/attributes mapped to a common object or category. The Y-axis represents the spatiotemporal disparity between the stimuli. The effects of crossmodal correspondences and semantic congruency often occur between stimuli in a larger temporal disparity over hundreds of ms that are represented as two distinct events, such as the studies demonstrating crossmodal semantic priming (Chen and Spence, 2011b, 2013). The unity effect attributable to crossmodal correspondences or semantic congruency has, though, only been observed when the stimuli were presented in a range within 100 ms (Vatakis and Spence, 2007; Parise and Spence, 2009).

Typically, research on the crossmodal correspondences, the latter referring to the compatibility between features or polarized dimensions between crossmodal stimuli (see Spence, 2011, for a review), has traditionally *not* been considered within the literature on the unity effect. However, it is clear that crossmodal correspondences can be seen as fitting within the broad scope of the unity assumption. Indeed, a growing body of research conducted over the last 40 years or so has shown that people feel that certain sensory-specific attributes (or features) go, or belong, together, even if they do not necessarily believe that they ever co-occur within one and the same object (see **Figure 2**). For example, even though a higher-pitched tone is likely produced by a relatively smaller object, the mapping between the dimensions of size and pitch is relative and context-dependent rather than absolute (i.e., there is no one-to-one mapping, see Gallace and Spence, 2006).

**FIGURE 3:**
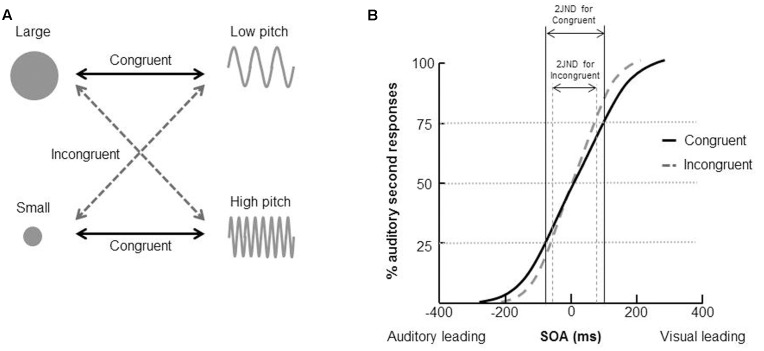
**Examples of stimuli and the results of Parise and Spence’s (2009) study of the unity effect using in a temporal order judgment (TOJ) task.** **(A)** The crossmodal correspondences between visual size and auditory pitch. **(B)** The results demonstrated that it was harder for participants to correctly judge the presentation order of a visual and auditory stimulus (i.e., the just noticeable difference (JND) was significantly higher) when the stimuli were congruent than incongruent.

Parise and Spence (2009), for example, demonstrated that people exhibited a significant unity effect attributable to crossmodal correspondences (see also Parise and Spence, 2008). In their study, participants made unspeeded temporal order judgments (TOJs) concerning whether a visual or auditory stimulus had been presented second^4^ (see the section “The Temporal Ventriloquism Effect”). The stimulus onset asynchrony (SOA) was varied on a trial-by-trial basis using the method of constant stimuli. The visual and auditory stimuli presented in each trial were chosen to be either crossmodally congruent or incongruent; in particular, the visual stimulus consisted of either a smaller or larger circle, corresponding to higher- or lower-pitched tones in the auditory modality, respectively (see **Figure 3A**). The results demonstrated that participants found it significantly harder to discriminate the correct temporal order of the visual and auditory stimuli (i.e., a larger just noticeable difference (JND) was observed) for those pairs of stimuli that were crossmodally congruent than for those pairs that were incongruent (see **Figure 3B**). A similar pattern of results was obtained from the correspondence between rounded visual shape and auditory low-pitched sine-wave tone, as well as between spikey visual shapes and auditory high-pitched square-wave tone.

What is more, Parise and Spence (2009) demonstrated that audiovisual crossmodal correspondences between visual size and auditory pitch also modulate the *spatial* aspects of multisensory integration. That is, crossmodally congruent pairs of visual and auditory stimuli gave rise to spatial ventriloquism over a wider range of spatial disparities than incongruent stimulus pairings. The results of the three experiments reported by Parise and Spence (2009) are consistent with the view that more pronounced multisensory integration occurs for congruent visual and auditory stimuli than for those pairs of stimuli that happen to be incongruent. Hence, research on the crossmodal correspondences provides support for the unity assumption (see also Miller, 1991, for a similar conclusion based on a study of the redundant target effect). However, here it is worth noting, in closing, that just because certain crossmodal correspondences influence multisensory integration, it certainly does not mean that all correspondences necessarily will (e.g., Stekelenburg and Keetels, 2015).

### Semantic Congruency

A large body of empirical research has demonstrated the influence on perception in one sensory modality of the presentation of a semantically congruent stimulus in another modality (e.g., a dog’s image and a barking sound), as compared to a semantically incongruent one. To date, the majority of such studies have focused on the case of audiovisual interactions (see Doehrmann and Naumer, 2008, for a review). Researchers often presume that visual and auditory stimuli are integrated when they are semantically congruent and some form of enhancement in their participants’ behavioral performance or brain activities will be observed (Laurienti et al., 2004; Molholm et al., 2004; Taylor et al., 2006; Hein et al., 2007; Yuval-Greenberg and Deouell, 2007; Adam and Noppeney, 2010; Chen and Spence, 2010; Werner and Noppeney, 2010).

Once again, it can be argued that the literature on semantic congruency effects can be subsumed within broader questions about the influence of the unity assumption on multisensory integration. Just as was the case for the literature on crossmodal correspondences, it is hard to tease apart the exact differences between those bodies of research. Over the years, however, the unity effects and semantic congruency effects have tended to be grouped under rather different research headings.

Here, it is also worth noting that Chen and Spence (2011b, 2013) did not actually consider the crossmodal semantic congruency effects they documented as resulting from multisensory integration. Rather, they suggested that these effects could simply be explained in terms of the semantic priming of one object/event by another (see Gordon and Irwin, 1996). Such an interpretation was supported by the fact that the semantic congruency effect appeared to be maximal when the auditory stimulus was presented a few hundreds of ms before the visual stimulus rather than when the two stimuli were presented at the same time. In addition, semantic congruency effects can be explained by memory consolidation whenever the auditory stimulus is presented some few hundreds of ms after the visual stimulus (Chen and Spence, 2010).

The majority of studies that have attempted to assess the impact of semantic congruency on multisensory perception have actually used stimuli that, while referring to the same basic *category* or *concept*, are not realistic objects or events that we encounter in daily life; that is, they often lack ecological validity. In fact, most studies of semantic congruency have presented line drawings or pictures of objects (the former often taken from the classic database of Snodgrass and Vanderwart, 1980) together with a sound that is typically produced by a given object – e.g., the meowing of a cat to go with the static picture of a cat, or a barking sound to go with the picture of a dog. Indeed, Edmiston and Lupyan (2015) have recently demonstrated that people’s matching performance between a picture and a sound was systematically improved when the picture resembled the moment when a sound is producing (e.g., a dog with open mouth^5^). As such, it can be argued that there would seem to be little likelihood that the participants in those studies using static line-drawings had any reason to believe that what they were seeing and hearing actually referred to the same object or event.

### Context

In an early study, Engel and Dougherty (1971) reported that people’s perception of audiovisual simultaneity is systematically shifted with the distance of the stimulus location (see also Sugita and Suzuki, 2003; Alais and Carlile, 2005; though see Arnold et al., 2005). Later studies demonstrated that, either after passively adapting to asynchronous audiovisual events (Fujisaki et al., 2004; Vroomen et al., 2004), or after being trained to discriminate audiovisual synchrony (vs. asynchrony) with feedback (Powers et al., 2009), people’s perception of audiovisual simultaneity would change accordingly (see Vroomen and Keetels, 2010, for a review). These results suggest that the mechanisms underlying human multisensory perception are flexible and malleable.

Such flexibility in multisensory perception leads to the contextual effect that, when encountering the same pairs of multisensory stimuli, the processing of these stimuli can be modulated by the context provided by prior perceptual experience. For example, people were more likely to integrate the visual and auditory signals after having been presented with congruent (rather than incongruent) pairs of visual lip movements and spoken syllables (Nahorna et al., 2012). The contextual modulation can be very rapid in the case of audiovisual perception, occurring even on a trial-by-trial basis. For example, when the asynchronous visual and auditory stimuli in a given trial were perceived as having been presented at the same time, this can bias the perception in the following trial (van der Burg et al., 2013). Hence, audiovisual perception seems highly malleable and susceptible to the context induced by prior experience. Nevertheless, similar results have not been observed in visuotactile or audiotactile perception yet (van der Burg et al., 2015).

### Interim Summary

In this section, the possible factors inducing the unity assumption have been reviewed: the unity assumption can either be provided explicitly, typically by means of instructions from the experimenter, or may emerge implicitly based on the properties of the stimuli that are presented (including redundant information, crossmodal correspondences, and semantic congruency). Researchers plausibly agree that the latter three serve as critical factors underlying the unity effect. Nevertheless these influences may (and mostly have) been examined as independent factors in the empirical literature. This fact, on the other hand, may also reflect the fact that, even though crossmodal stimuli are inherently associated when they are redundant, corresponding, or semantically congruent, they do not necessarily have to be integrated as a unitary object or event representation and lead to the unity effect. Finally, prior experience provides a context that modulates the subsequent multisensory stimuli to be integrated, or kept separate instead.

## Empirical Evidence of the Unity Effect

Reviewing the literature in this area, it soon becomes apparent that the unity effect has proved to be one of the most contentious issues in multisensory perception research over the last 60 years or so (e.g., Vroomen, 1999; Welch, 1999; see Welch and Warren, 1980; Vatakis and Spence, 2007; Chen and Vroomen, 2013, for reviews). Below, we review the evidence of the unity effect from four paradigms: spatial and temporal ventriloquism, the McGurk effect, and the Colavita visual dominance effect. These paradigms are commonly used to evaluate the unity effect because they are conventionally considered as prototypical examples of multisensory integration.

### The Spatial Ventriloquism Effect

The first empirical evidence relevant to addressing the unity effect was published by Jackson (1953; see **Table 1**). He used spatial ventriloquism whereby judgments of the location from which a sound had been presented were biased by a spatially disparate visual stimulus. The unity effect was demonstrated by the bias in the perceived location of the sound of a steam whistle resulting from the sight of a steaming kettle being larger than the bias of a bell sound that was paired with a spark of light. The latter was an arbitrary combination of auditory and visual stimuli that should not have led to a strong assumption of unity. These results have been taken by some researchers to suggest that any unity assumption that results from a semantically congruent (as opposed to incongruent) stimulus pair can indeed facilitate multisensory integration across a wider range of spatial discrepancies. An alternative possibility here, though, is that the temporal correlation between the whistle and kettle signals, given their rich temporal variation, was presumably higher than the pairing of the bell and the spark of light. This proposition is supported by recent findings demonstrating that temporally correlated signals do indeed give rise to an increase in multisensory binding (e.g., see Parise et al., 2012, 2013). In summary, the suggestion is that the strength of the coupling between the visual and auditory stimuli in terms of their semantic congruency and/or temporal correlation modulates the unity effect as indexed by the disparity range over which spatial ventriloquism occurred in Jackson’s (1953) study.

**Table 1:** Summary of the unity effect demonstrated in studies of the spatial ventriloquism effect.

Study	Origins of the unityassumption	Stimuli	Experimental paradigm	Effect?
Jackson, 1953	Semantic congruency, redundant information (temporal structure)	Kettle and whistle vs. light and bell	Spatial ventriloquism	Yes
Warren et al., 1981	Instruction, redundant information (temporal synchrony), and semantic congruency	Human face and voice vs. tape mark and voice (Experiment 1)	Spatial ventriloquism (Experiment 1)	Yes, but only when the stimuli were synchronous and semantically congruent


		Human face/spot and voice/click (Experiment 4)	Spatial discrimination (Experiment 4)	
Wallace et al., 2004	Redundant information (spatial and temporal coincidence)	Light and white noise	Spatial ventriloquism	Yes
Parise and Spence, 2009	Crossmodal correspondence (size and pitch)	Visual disk and pure tone	Spatial discrimination	Yes
Kanaya and Yokosawa, 2011	Semantic congruency	Human speech	Spatial ventriloquism	Yes
Wozny and Shams, 2011	Context	Visual white-noise disk and auditory white-noise burst	Auditory spatial realignment	Yes
Radeau and Bertelson, 1977	Semantic congruency	Human speech or playing bongos (full video vs. synchronized light)	Spatial ventriloquism aftereffect	No
Radeau and Bertelson, 1978	Semantic congruency, instruction	Playing bongos (full video vs. synchronized light)	Spatial ventriloquism aftereffect	No
Colin et al., 2001	Semantic congruency	Human speech	Spatial ventriloquism	No


In contrast to Jackson’s (1953) results, though, other researchers subsequently failed to demonstrate any unity effect when using the spatial ventriloquism aftereffect as the dependent variable. For example, Radeau and Bertelson (1977, 1978) presented their participants with realistic audiovisual pairings, such as the video of a person’s speaking face and voice, or the video of the hands of someone playing the bongos and the associated drumming sounds. For comparisons, non-realistic pairings consisted of the same sounds but the visual stimuli were replaced by a light that was synchronized with the rhythm of the sounds. The influence of the unity assumption was assessed by measuring the change in unisensory auditory localization performance following adaptation to the auditory and visual stimuli that had been separated by 20°. Specifically, the spatial representation of the auditory stimuli should be re-aligned toward the location of the visual stimulus if they were integrated during adaptation. Such audiovisual spatial re-alignment would remain after adaptation, therefore named the *ventriloquism aftereffect*.

Radeau and Bertelson’s (1977, 1978) results revealed that the magnitude of the ventriloquism aftereffect was similar following adaptation to both realistic and unrealistic stimulus pairings. These similar aftereffects, though, can perhaps be attributed either to the particular stimuli that were used or to the specific experimental paradigm. Note that the visual and auditory stimuli in both the realistic and unrealistic pairings were highly correlated in terms of their temporal structure. This might have been sufficient to lead to multisensory integration regardless of the realism of the stimuli (e.g., Parise et al., 2012, 2013)^6^. In addition, in their test session following adaptation, only the to-be-localized sound, rather than any visual stimulus, was presented (the participants were, in fact, blindfolded). Hence, it could perhaps be argued that the unity assumption is constructed online when multisensory stimuli are presented and simply did not carry-over to the following unisensory test session.

**FIGURE 4:**
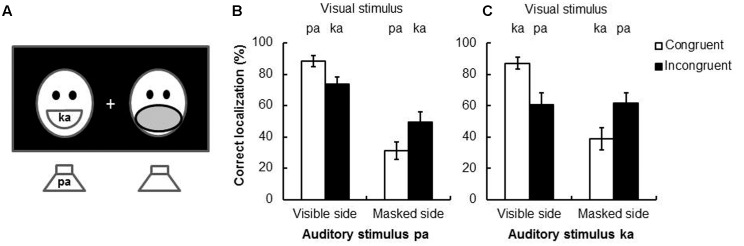
**The experimental setting and results of Kanaya and Yokosawa’s (2011) study looking at the McGurk effect in a spatial ventriloquism paradigm.** **(A)** The experimental setting of the study. Two human faces were presented side-by-side, one was intact and the other was masked. Two speakers were put below and aligned with the location of the faces. **(B)** The results for the auditory stimulus *pa*. In the incongruent condition (i.e., hearing *pa* but seeing *ka*) led to the perception of *ta* (i.e., the McGurk effect). In this case, spatial ventriloquism may still occur; that is, the sound localization performance was less accurate when it was presented at the masked side than the visible side. **(C)** The results of auditory stimulus *ka*. In the incongruent condition (with visual stimulus *pa*), no McGurk effect would occur; in this case, spatial ventriloquism did not occur either. That is, sound localization performance was similar when it was presented at the masked or visible side. **(B,C)** reproduced from Kanaya and Yokosawa (2011) with data provided by the authors.

When using speech stimuli in the condition where the visual stimulus was presented together with the to-be-localized sound, inconsistent results were nevertheless still observed. For example, Colin et al. (2001) reported that the magnitude of the spatial ventriloquism effect was unaffected by the congruency between visual and auditory speech syllables. On the other hand, a very different pattern of results was reported by Kanaya and Yokosawa (2011): they conducted a study of the spatial ventriloquism effect in which a fully visible face was presented on one side of fixation and a face with a mask on the mouth on the other (see **Figure 4A**). Each face uttered one syllable, either /ka/ or /pa/. At the same time, /ka/ or /pa/ was presented auditorily from a loudspeaker situated on the bottom left or right of the screen on which the faces were presented. The participants had to judge the side (left vs. right) from which the speech sound appeared to have originated. The results demonstrated that visual capture primarily occurred in response to the fully visible face (i.e., a lower sound localization accuracy was observed on the masked side than on the visible side, see **Figure 4B**). However, over-and-above this basic effect of visual saliency, a significantly larger ventriloquism effect was also documented when the visible face and the voice uttered the same syllable (the difference between the two white bars was 57.2%) than when they uttered different syllables (the difference between the two black bars was 24.5%).

Even though the spatial ventriloquism effect has been used to demonstrate the unity effect ever since Jackson’s (1953) seminal paper, there is a question mark here as to whether spatial ventriloquism is, in fact, a valid experimental paradigm. The question that crops up here emerges from a closer inspection of Welch and Warren’s (1980) early model. Specifically, according to their conceptualization (see **Figure 1**), the spatial and temporal structure of the incoming sensory stimuli are analyzed prior to the formation of the unity assumption. As such, one might wonder why the unity effect should be indexed by the modulation of the size of the window of the spatial ventriloquism effect. Alternatively, however, one might imagine that such a unity effect was simply a result of response bias induced by the presence of the congruent visual signal instead (see Choe et al., 1975; Bertelson and Radeau, 1981).

**FIGURE 5:**
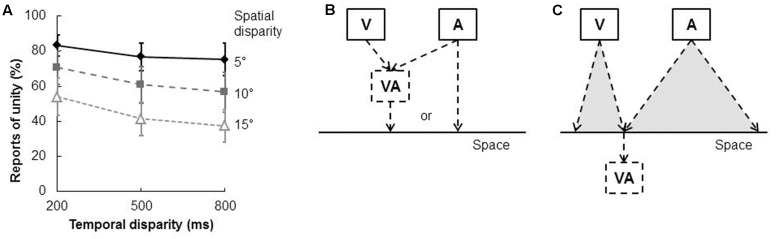
**Results of Wallace et al.’s (2004) study of the unity effect and the spatial ventriloquism effect.** **(A)** In the unification judgment task (i.e., judging whether the visual and auditory stimuli were presented from the same or different locations), the proportion of unification judgments decreased when either spatial or temporal disparity increased. **(B)** Hypothesis 1 suggests that the judgment of auditory localization occurs after whether visual and auditory signals were integrated (i.e., unified) in the spatial domain. That is, the spatial ventriloquism effect results from a unified percept. The auditory localization should be quite accurate if the visual and auditory signals were not integrated. **(C)** Hypothesis 2 suggests that the unification judgment is determined by the perceived location of each visual and auditory signal. That is, the visual and auditory inputs would be judged as unified if they happened to be perceived at the same location. **(A)** Reproduced from the data provided in Wallace et al. (2004).

Wallace et al. (2004) verified that the spatial ventriloquism effect is correlated with judgments of unification. These researchers manipulated both the spatial and temporal disparity between the visual and auditory stimuli (in this case, an LED and a burst of white noise) that were presented to participants. The latter had to try and localize the sound as well as to make a judgment concerning whether the visual and auditory stimuli appeared to have been presented from the same location or not (i.e., they had to make a judgment concerning the unification of the stimuli). As might have been expected, the proportion of unification judgments decreased as the spatial and temporal disparity between the visual and auditory stimuli increased (see **Figure 5A**). Interestingly, the proportion of unification judgments was also positively correlated with the magnitude of the spatial ventriloquism effect in the sound localization task (see Figure 2 in Wallace et al., 2004). However, when the visual and auditory stimuli were not judged as unified, either no bias or else a small repulsion effect was observed instead. That is, the sound was more likely to be localized toward the side opposite to the light (that is, a counter-ventriloquism effect was obtained; see also Körding et al., 2007; Rohe and Noppeney, 2015b).

It is important to note, when thinking about these results, that it is hard to distinguish between two possible causal relations that might potentially have given rise to the observed correlation: according to one hypothesis (see **Figure 5B**), the spatial ventriloquism effect simply resulted from audiovisual integration (i.e., the visual and auditory stimuli were unified). It is, though, hard to explain the counter-ventriloquism effect that was observed with this account; namely, one might have expected that sound localization performance was accurate in the absence of audiovisual integration (i.e., in the absence of unification). The existence of the counter-ventriloquism effect might suggest that the sound localization judgment can simply be considered as reflecting a response strategy that follows on from the unification judgment instead.

According to a second hypothesis (see **Figure 5C**), the unification judgment is based on the perceived location of the visual and auditory stimuli as being either the same or different. That is, the unification response can be made even when visual and auditory stimuli were represented as two distinct unisensory events that just happened to be presented from the same location. According to this view, counter-ventriloquism can be explained as an error of unisensory auditory localization.

Support for the first hypothesis comes from the results of a study by Wozny and Shams (2011). These researchers utilized audiovisual spatial recalibration on a trial-by-trial basis to probe the influence of audiovisual integration. The hypothesis here is that if the visual and auditory stimuli in the preceding trial happened to have been integrated and represented as a single event, then their spatial disparity would be recalibrated and thus minimized. Presumably, if such a recalibration effect is carried-over to the following unisensory auditory trial, the perceived location of the sound in that trial would be realigned toward the location of the visual stimulus in the preceding trial. The results indeed demonstrated a causal relation between audiovisual integration and auditory spatial realignment. In particular, the spatial realignment of auditory localization in a given trial was more pronounced when the visual and auditory stimuli in the preceding trial were perceived as unified (defined operationally as their perceived spatial disparity being smaller than 0.5°) than when they were not (defined as when their perceived spatial disparity was larger than 6°).

Additional evidence in support of the suggestion that the unity assumption leads to a more pronounced spatial ventriloquism effect comes from the aforementioned study by Kanaya and Yokosawa (2011). Specifically, one of the incongruent stimulus combinations (auditory /pa/, visual /ka/) likely gave rise to the McGurk effect (perceived /ta/, see the section “The McGurk Effect”), whereas the other pairing (auditory /ka/, visual /pa/) did not. Hence, incongruent syllables were presented visually and auditorily in both conditions, while the perception in the former condition was likely to have been unified (i.e., the pairing giving rise to the McGurk effect) but not in the latter. A larger spatial ventriloquism effect was observed for McGurk stimulus pairs that could be unified (in **Figure 4B**, the differences between the two black bars was 24.5%) than the other that could not (in **Figure 4C**, the differences between the two black bars was -0.8%). These results therefore suggest that the unity assumption gives rise to enhanced audiovisual integration (i.e., a more pronounced spatial ventriloquism effect)^7^.

### The Temporal Ventriloquism Effect

**FIGURE 6:**
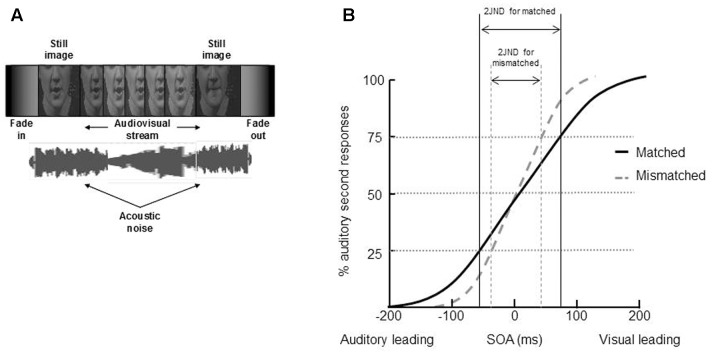
**An example of stimuli and results of Vatakis and colleagues’ experiments of the unity effect on temporal perception (Vatakis and Spence, 2007, 2008; Vatakis et al., 2008).** **(A)** An example of the video and audio used in the studies – they were either matched or not in terms of gender. **(B)** The results demonstrated that it was harder for participants to correctly judge the presentation order of the video and audio (i.e., the JND, was significantly higher) when the stimuli were matched than mismatched. **(A)** Reprinted from Vatakis and Spence (2008) with permission from the authors.

The unity effect has been demonstrated not only in the spatial, but also in the temporal domain (see **Table 2**). So, for example, the participants in a series of four audiovisual TOJ experiments conducted by Vatakis and Spence (2007) were presented with pairs of auditory and visual speech stimuli (either single syllables or words) at various SOAs. The participants in this study had to make unspeeded TOJs regarding whether the auditory or visual speech stream had been presented first on each trial. On half of the trials, the auditory and visual speech stimuli were gender matched (i.e., a female face was presented together with a female voice), while on the remainder of the trials, the auditory and visual speech stimuli were gender mismatched (i.e., a female face was presented together with a man’s voice; see **Figure 6A**). The participants in all four of Vatakis and Spence’s experiments found it significantly harder to judge which modality had been presented first when evaluating the matched stimuli (JND = 89 ms) than the mismatched stimuli (JND = 68 ms; these values reflect the average JNDs across Vatakis and Spence, 2007, Experiments 1–3; see **Figure 6B**).

**Table 2:** Summary of the unity effect demonstrated in studies of the temporal ventriloquism effect.

Study	Origins of the unityassumption	Stimuli	Experimental paradigm	Effect?
Vatakis and Spence, 2007	Semantic congruency	Human speech	TOJ	Yes
Vatakis et al., 2008	Semantic congruency	Human speech, monkey calls	TOJ	Yes, but only for human speech
Parise and Spence, 2008	Crossmodal correspondence (size and pitch)	Visual disk and pure tone	Temporal ventriloquism	Yes
Parise and Spence, 2009	Crossmodal correspondence (size and pitch; shape and pitch)	Visual disk and pure tone, Visual pattern and pure tone	TOJ	Yes
Vatakis and Spence, 2008	Semantic congruency	Playing instruments and object actions (hammer smash ices and ball bouncing)	TOJ	No
Keetels and Vroomen, 2011	Crossmodal correspondence (size and pitch)	Visual disk and pure tone	Temporal ventriloquism	Replicated the condition in Parise and Spence (2008), but the result cannot explained by the temporal ventriloquism effect


*TOJ, temporal order judgments.*

Vatakis and Spence (2007) suggested that the presentation of the matched speech stimuli may have resulted in more temporal ventriloquism (e.g., Morein-Zamir et al., 2003) than was the case for the mismatched stimuli (see also Parise and Spence, 2008; though see Keetels and Vroomen, 2011). That is, the visual event was temporally aligned to the slightly asynchronous auditory event, and the unity assumption result in this temporal alignment occurring over even wider range of intervals in the gender-matched (as compared to the gender-mismatched) condition. These results therefore provide empirical support for the claim that the unity assumption can enhance the integration of visual and auditory speech stimuli in the temporal domain.

However, subsequent research has complicated the story somewhat. In particular, while Vatakis et al. (2008) replicated the unity effect for audiovisual speech stimuli, they repeatedly (across seven separate experiments) failed to demonstrate any unity effect when their participants were presented with matched vs. mismatched videos of musical stimuli (the two stimuli in this case being a person playing a note on a piano vs. on a guitar), object actions (someone smashing a block of ice or dropping a ball on the ground), or monkey calls (monkeys recorded making either a cooing or a grunting noise) instead (Vatakis and Spence, 2008; Vatakis et al., 2008). Such results led Vatakis and Spence to conclude, in line with previous researchers, that human speech stimuli might, in some sense, be special.

Before accepting the undoubtedly controversial claim that speech really is ‘special’ (see also Saldaña and Rosenblum, 1993; Tuomainen et al., 2005; though see Rosenblum, 2008; Vroomen and Stekelenburg, 2011), a number of alternative explanations for these null results need to be ruled out first. One might worry, for instance, that the participants simply did not notice the discrepancy between what they heard and saw in the mismatched condition and hence all of the audiovisual stimulus displays might just have induced a similar assumption of unity. However, a number of control experiments conducted by Vatakis and Spence (2008) revealed that their participants were near-perfect when it came to discriminating between the congruent and incongruent audiovisual stimuli. A second possible alternative explanation might simply involve the suggestion that we have all had much more exposure to audiovisual speech stimuli than to musical stimuli or animal vocalizations, and hence the unity effect might just need very large amounts of prior experience in order to be demonstrated. Indeed, sometimes experts are more sensitive to audiovisual asynchrony than novices (e.g., Petrini et al., 2009; Lee and Noppeney, 2014). However, control experiments with trained musicians and those working extensively with monkeys once again failed to demonstrate a unity effect with stimuli in their area of expertise/familiarity that the participants could easily segregate into matched vs. mismatched stimulus pairs.

### Interim Summary Concerning the Spatial and Temporal Ventriloquism Effect

The literature reviewed in the above two sections has demonstrated that the unity assumption enhances multisensory integration in terms of a more pronounced spatial or temporal ventriloquism effect under certain conditions. Nevertheless, in reviewing these studies, a number of pitfalls associated with early attempts to provide evidence in support of the unity assumption have also been highlighted. For example, while the spatial ventriloquism effect is modulated by the unity assumption (e.g., Kanaya and Yokosawa, 2011; Wozny and Shams, 2011), such unequivocal evidence for the temporal ventriloquism effect using the TOJ task is currently lacking. In addition, while the unity effect has been demonstrated reliably using human speech stimuli, it does not necessarily extend to the case of other classes of stimuli, such as audiovisual music clips (see Vatakis and Spence, 2007, 2008; Vatakis et al., 2008; though see Jackson, 1953).

### The McGurk Effect

According to the argument that speech is special, one should also expect to find a unity effect for other examples of audiovisual speech integration, such as the McGurk effect (McGurk and MacDonald, 1976). Previous research has manipulated various factors that may lead to different levels of the unity assumption, such as spatial and temporal disparities, stimulus congruency, and context (see **Table 3**).

**Table 3:** Summary of the unity effect demonstrated in studies of the McGurk effect.

Study	Origins of the unityassumption	Stimuli	Perception	Effect?
Massaro and Cohen, 1993	Temporal synchrony (±200 ms)	V: /da/; A: /ba/	/va/ or /ga/	Yes: /va/ decreased and /ga/ increased when V leading
Munhall et al., 1996	Temporal synchrony (±360 ms)	V: /aga/ or /igi/; A: /aba/	/ada/ or /idi/	Yes: -60 to 240 ms for V/aga/
	Stimulus congruency (vowel)			No effect for V/igi/
van Wassenhove et al., 2007	Temporal synchrony (±467 ms)	V: /ka/ or /ga/; A: /pa/ or /ba/	/ta/ or /da/	Yes: -30 to 170 ms
Soto-Faraco and Alsius, 2009	Temporal synchrony (-640 to 720 ms)	V: /ba/; A:/da/	/bda/	Yes: -320 to 480 ms
Jones and Munhall, 1997	Spatial disparity (±90°)	V: /igi/, /IgI/ or /ægæ/	/idi/, /IdI/ or /ædæ/	No
		A: /igi/, /IgI/ or /ægæ/		
Jones and Jarick, 2006	Temporal synchrony (±360 ms)	V: /ava/; A: /aba/	/ava/ or /aba/	Yes: -60 to 180 ms
	Spatial disparity (±90°)			No effect for spatial disparity
Easton and Basala, 1982	Congruency (phonetic)	V: lips movements; A: spoken words	Errors in lip-reading	Yes: fewer errors in lip-reading in the higher discrepancy condition
Green et al., 1991	Congruency (gender)	V /ga/ or /gi/; A:/ba/ or /bi/	/da/, /ða/ or /di/, /ði/	No
Walker et al., 1995	Familiarity (face and voice from familiar or unfamiliar person)	V /ga/ or /gi/; A:/ba/ or /bi/	/da/, /ða/ or /di/, /ði/	Yes: the McGurk effect was larger for familiar face and voice
Nahorna et al., 2012	Context (coherence of audiovisual syllables)	V: /ga/; A:/ba/	/da/	Yes: larger McGurk effect in the coherent context
Nahorna et al., 2015	Context (coherence of audiovisual syllables)	V: /ga/; A:/ba/	/da/	Yes: smaller McGurk effect when perceiving one incoherent syllable, but recovered after perceiving more coherent syllables


*V: vision; A: audition.*

*The negative values indicate the auditory leading intervals, whereas the positive values indicate the visual leading intervals; the negative angles indicate that the auditory stimulus was presented on the left, whereas the positive angles indicate that the auditory stimulus was presented on the right.*

Temporal synchrony and spatial coincidence, the two basic rules of multisensory integration, have been tested in the McGurk effect. The McGurk effect is reliably observed in a temporal window which is asymmetrical. Specifically, this window is wider in the condition where the visual leading auditory stimulus than *vice versa* (Massaro and Cohen, 1993; Munhall et al., 1996; Jones and Jarick, 2006; van Wassenhove et al., 2007; Soto-Faraco and Alsius, 2009)^8^. By contrast, the McGurk effect seems not to be influenced by the spatial disparity between the visual and auditory stimulus (Jones and Munhall, 1997; Jones and Jarick, 2006; see Spence, 2013, for a review).

In one of the above studies, Munhall et al. (1996) also presented the visual and auditory consonant that could lead to the McGurk effect being sandwiched between either matched or mismatched vowels. Specifically, their participants heard /aba/ while viewing the lip movements associated with /aga/ (matched vowels) or /igi/ (mismatched vowels). The results revealed that the McGurk effect (i.e., perceiving the consonant as /d/) was larger when the auditory and visual vowels were matched.

Meanwhile, Easton and Basala (1982) conducted two experiments in which they assessed the ability of participants to lip-read (monosyllabic or compound words) under conditions of unisensory visual presentation vs. discrepant audiovisual presentation. In the latter condition, they varied the degree of discordance of the initial and/or final phonemes of the words that were presented. The participants’ recognition of visual speech (that is, their lip-reading performance) was substantially biased by the presence of discrepant auditory information (indexed by the error of participants misreporting the ‘lipped’ word as the dubbed word). Interestingly, this auditory bias decreased when both the initial and final phonemes (as compared to when only one of them) were discrepant. In addition, Easton and Basala also manipulated the gender of the speaker and the dubbed voice to either match or not. The result demonstrated that the auditory bias was smaller when the gender was mismatched (though see Green et al., 1991).

The matching between human faces and voices can nevertheless influence the McGurk effect in a more specific way as a function of familiarity. Walker et al.’s (1995) study demonstrated that the unity assumption may vary from one individual to the next. In particular, when participants reported being familiar with either the speaker’s face or voice, a reduced McGurk effect was observed when the speaker’s face or voice was replaced by another person’s, irrespective of whether they had the same or different gender. This result suggests that unity assumption can be formed for a particular pair of face and voice belonging to a person due to familiarity.

Finally, the McGurk effect is influenced by context (concerning stimulus congruency) that leads to the tendency of either binding the incoming audiovisual signals or not. For example, people demonstrated a larger McGurk effect if they had heard a series of audiovisual speech stimuli that were congruent rather than incongruent (Nahorna et al., 2012). Later studies demonstrated that such a contextual effect is highly malleable. Specifically, the tendency to separate visual and auditory stimuli can be rapidly established by just perceiving one incongruent audiovisual syllable, while it can also be reversed by experiencing more congruent audiovisual syllables (Nahorna et al., 2015). Such a contextual effect on segregating visual and auditory stimuli in the incongruent context is associated with increased activities at the left inferior frontal sulcus (Gau and Noppeney, 2016).

In summary, the temporal, rather than spatial, proximity between the visual and auditory stimuli has been shown to modulate the McGurk effect (e.g., Jones and Jarick, 2006). In addition, the congruency of stimulus identity modulates the extent to which people integrate multisensory speech stimuli (Easton and Basala, 1982; Munhall et al., 1996; though see Green et al., 1991). Furthermore, familiarity with the speakers in the video-clips is also a strong modulatory factor at the level of the individual participant (see Walker et al., 1995). Finally, prior experience regarding whether the visual and auditory speech signals are congruent or not provides a context that modulates the magnitude of the McGurk effect.

### The Colavita Visual Dominance Effect

The Colavita effect is the name that has been given to an example of visual dominance over audition (see Colavita, 1974). In a typical study, participants are presented with an unpredictable sequence of visual, auditory, and audiovisual targets requiring a speeded detection response. Oftentimes, the participants are instructed to press one response key whenever the visual target is presented and another key whenever the auditory target is presented; on the bimodal trials, the participants are instructed to press both response keys (or else to press a third key). No matter how the participants respond, a common result that has been obtained over the years is that the participants fail to respond to some proportion of the auditory targets on the bimodal trials (i.e., they only respond to the visual target), while making very few errors on the unimodal auditory trials (see Spence et al., 2011, for a review). In fact, it is as if the simultaneous presentation of the visual stimulus extinguishes the participant’s awareness of, or at least their ability to respond to, the auditory stimulus on a certain proportion of the bimodal trials.

A partial answer concerning whether the unity assumption modulates the Colavita visual dominance effect has come from a series of experiments reported by Spence and his colleagues (see **Table 4**). For example, both temporal synchrony and spatial coincidence factors modulate the Colavita effect (defined as the increased likelihood of missing the auditory target than missing the visual target on the bimodal trials). Koppen and Spence (2007a) manipulated the SOA between the visual and auditory targets on the bimodal trials. The results demonstrated the Colavita effect was observed over the window from when auditory led by 35 ms through until visual leading by 150 ms. Similarly, the Colavita effect occurred more often when the visual and auditory targets were presented at the same location than from different locations (13° or 26° disparity) on the bimodal trials (Koppen and Spence, 2007b; see Hartcher-O’Brien et al., 2008, for the Colavita effect showing vision’s dominance over touch too). In summary, on the bimodal trials, the visual and auditory targets that are presented close in time and space led to a larger Colavita effect.

**Table 4:** Summary of the unity effect demonstrated in studies of the Colavita visual dominance effect.

Study	Origins of the unityassumption	Stimuli	Experimental paradigm	Effect?
Koppen and Spence, 2007a	Temporal synchrony (±600 ms)	V: LED; A: pure tone (4000 Hz)	Speeded detection	Yes: -35 to 150 ms
Koppen and Spence, 2007b	Spatial disparity (±13° or ±26°)	V: LED; A: white noise	Speeded detection	Yes: larger Colavita effect in the same location condition
Hartcher-O’Brien et al., 2008	Spatial disparity (±12.5°)	V: LED; T: tactile vibrations	Speeded detection	Yes: larger Colavita effect in the same location condition
Koppen et al., 2008	Semantic congruency	V: dog or cat picture; A: barking or meowing sound	Speeded detection	No
Stekelenburg and Keetels, 2015	Crossmodal correspondence (size and pitch)	Visual disk and pure tone	Speeded detection	No


*V: vision; A: audition; T: touch.*

*The negative values indicate the auditory leading intervals, whereas the positive values indicate the visual leading intervals; the negative angles indicate that the auditory stimulus was presented on the left, whereas the positive angles indicate that the auditory stimulus was presented on the right.*

A third factor eliciting the unity assumption that has been tested in the Colavita effect literature is semantic congruency. The auditory stimuli in this study consisted of the sound of a cat meowing or a dog woofing, and the visual stimuli consisted of the pictures of a cat and of a dog (Koppen et al., 2008). On the bimodal trials, the auditory and visual stimuli could either be semantically congruent (i.e., the sight and sound of a dog) or else semantically incongruent (i.e., the sound of a cat presented together with the sight of a dog). The magnitude of the Colavita visual dominance effect was completely unaffected by the semantic congruency between the auditory and visual stimuli. This result was also replicated when using audiovisual speech stimuli in their Experiment 3.

Importantly, however, Koppen et al. (2008) found that the semantic congruency between the visual and auditory stimuli influenced certain other aspects of participants’ performance when the bimodal trials were associated with a third response key. Specifically, reaction times on the bimodally congruent trials were significantly faster than on the bimodally incongruent trials, a result that can perhaps best be explained in terms of the effect of semantic congruency on the redundant targets effect (see Miller, 1991; Laurienti et al., 2004). Hence, semantic congruency only influenced the participant’s response when both visual and auditory stimuli were processed (i.e., the participants correctly pressed the key corresponding to perceiving both visual and auditory stimuli). This result therefore suggests that the Colavita visual dominance effect may occur at an earlier stage of information processing than the stage at which crossmodal semantic congruency is computed (see Spence et al., 2011, for a review).

Stekelenburg and Keetels (2015) tested whether the crossmodal correspondence between visual size and auditory pitch (i.e., larger size matched to lower-pitched sounds) would modulate the Colavita visual dominance effect. Once again, a similar Colavita effect was observed in the matched and mismatched conditions. Furthermore, they did not observe any modulation of reaction times as a function of the crossmodal correspondence between the stimuli. One explanation for this is that the detection of stimulus congruency (around 400 ms after stimulus onset in terms of their event-related potentials results) was later than the decision and/or planning of motor responses (note that the participants’ mean reaction time was 640 ms on the bimodal trials). An alternative possibility here is that it may be hard to elicit any crossmodal correspondence effects implicitly, unless the participants are told (or aware of) the relationship between the component stimuli (e.g., Klapetek et al., 2012).

The results reviewed in this section demonstrate that the Colavita visual dominance effect follows the spatial and temporal rules of multisensory integration. However, the unity assumption, either induced by semantic congruency or by crossmodal correspondences, cannot modulate the magnitude of the Colavita effect (Koppen et al., 2008; Stekelenburg and Keetels, 2015). Participants’ performance in terms of the reaction time measure was, nevertheless, susceptible to semantic congruency when visual and auditory stimuli were both detected (Koppen et al., 2008). Combining these results suggests that temporal synchrony and spatial coincidence factors may modulate the Colavita effect in a bottom–up manner (see the model shown in **Figure 1**). The weak influence of the unity assumption in the Colavita effect suggests that the visual dominance likely occurs at an early stage of information processing, and so the unity assumption (either based on crossmodal correspondences, or semantic congruency) cannot penetrate down to this stage.

## From the ‘Unity Assumption’ to Bayesian ‘Priors’

Thus far, we have reviewed the empirical evidence concerning the unity effect by comparing those multisensory stimulus pairs of which the observer has a reason to believe ought to go together to the other pairs that were either mismatched or unrelated. At around the same time as Vatakis and Spence (2007, 2008; Vatakis et al., 2008) were revisiting the unity effect in human behavior, other researchers were examining this issue using the computational modeling approach based on Bayes’ rule (e.g., Ernst, 2007; Körding et al., 2007; see Shams and Beierholm, 2010, 2011, for reviews). According to such models, the unity assumption can be computed as a prior term.

### The Prior in the Bayesian Causal Inference Model

When two sensory inputs come from different modalities, our perceptual system may have to compute and infer whether they have a common cause (so that they should be integrated) or else different causes (in which case they should be kept separate). For example, in an audiovisual spatial ventriloquism experiment, if the participants infer that the two stimuli have a common cause, spatial ventriloquism should occur; otherwise, the stimuli are assumed to refer to different sources, and therefore no spatial ventriloquism effect is observed (e.g., Wallace et al., 2004). Such a causal inference process in the case of spatial ventriloquism was modeled using a Bayesian probability algorithm by Körding et al. (2007). In Körding et al.’s model, the variables include the perceived spatial locations of the visual and auditory stimuli, as well as a prior term (called *p*_common_ in the paper) denoting the observer’s knowledge as to how likely the two stimuli are to have a common cause. In the latest study reported by Odegaard and Shams (2016), it has been shown that the prior of common cause indeed positively correlated with the degree of multisensory integration. Furthermore, this prior is stable over time for a given participant in a given task. The Bayesian causal inference model has been used to study human behavioral responses (Wozny et al., 2010; Rohe and Noppeney, 2015b) and the underlying neural networks (Rohe and Noppeney, 2015a, 2016) when perfoming a spatial ventriloquism task.

Note that the prior term and the sensory inputs (or representations) in the Bayesian causal inference model are dissociated. So, for example, when the sensory representations change, such as the reliability of the stimulus being reduced by decreasing the luminance contrast of the visual stimulus, the prior remains constant (Beierholm et al., 2009). To date, however, only a few studies have empirically examined the precise value of the prior of common cause in the Bayesian causal inference model (e.g., Beierholm et al., 2009; Odegaard and Shams, 2016), while the question of how the prior systematically changes with the manipulated unity assumption is still unclear. For example, in Jackson’s (1953) classic demonstration of the unity effect in the spatial ventriloquism effect, the prior of common cause for the stimulus pairing of a steaming kettle and a whistling sound should be higher than that for the pair of light and bell; on the other hand, the spatial representations of the visual and auditory stimuli in the two conditions might be the same.

One might wonder what exactly the “prior” means here. In an early paper, Shams et al. (2005) modeled an audiovisual integration phenomenon called the sound-induced flash illusion (Shams et al., 2000) and proposed that the prior denotes “*…the perceptual knowledge of the observer about the auditory-visual events in the environment. In addition to the observer’s experience, the priors may also reflect hardwired biases imposed by the physiology and anatomy of the brain (e.g., the pattern of interconnectivity between the sensory areas), as well as biases imposed by the task, the observer’s state, etc.*” (Shams et al., 2005, p. 1924). Therefore, it would seem hard to characterize an observer’s priors as being attributable to the hard-wired neural connections which should be stable over time (Odegaard and Shams, 2016), or induced by a given set of environmental stimuli or conditions, while only the latter is relevant to the observer’s assumption or belief of unity. Another piece of evidence comes from the more pronounced effect of audiovisual integration in the peripheral as compared to central visual field (e.g., Charbonneau et al., 2013; Gleiss and Kayser, 2013; Chen et al., submitted), which is thought to be partly attributable to the more extensive neural connectivity across sensory-dominant areas in the periphery (Falchier et al., 2002; Rockland and Ojima, 2003).

In summary, researchers using the Bayesian causal inference model have successfully demonstrated that human performance in the spatial ventriloquism task is statistically optimal. Specifically, when determining whether visual and auditory signals would be integrated or separated, human brains compute sensory information as well as include a prior that represents a probability or tendency to integrate. In order to further link the Bayesian causal inference model to psychological mechanisms, one future goal would obviously be to examine whether the prior of common cause can be used to quantify the different levels of the unity assumption and to predict human behavior. In this case, a particular prior term may be able to match to a particular source of unity assumption as discussed in the section “Factors Leading to the Unity Assumption” (see Jones and Love, 2011).

### How are Priors Established?

It is clear that we can acquire new crossmodal associations between pairs of stimuli that have not been experienced as systematically related before. To put things simply, just imagine the situation of someone who has never seen a light saber before (as made famous by the Star Wars movies). On first seeing such a weapon, the person will presumably have no idea about what that weapon would sound like, or even that it should make a sound. Now, by repeatedly seeing and hearing the light saber in action, the person will presumably have sufficient evidence to establish knowledge of the light saber by the end of the movie. The key question here becomes how much exposure is required in order to establish or change the strength of the assumption of unity (or prior) – one may imagine that a relatively small number of exposures might be sufficient.

The available evidence from behavioral studies suggests that the crossmodal facilitation between arbitrary pairings of visual and auditory stimuli (such as letters and pure tones) is only observed when the sound and the visual target reliably co-occur during the course of the experiment (Lippert et al., 2007; Chen and Spence, 2011a). Neuroimaging evidence shows increased cross-cortical activation following even a very small number of co-occurrences (Fiebelkorn et al., 2010, 2012; Zangenehpour and Zatorre, 2010; Liu et al., 2012). The evidence from the world of olfactory-gustatory correspondences research suggests that such associations can be acquired after only a handful of trials when people are exposed to a novel odorant (see Stevenson and Boakes, 2004, for a review).

In daily life, the time required to learn new associations between multisensory stimuli (or to establish a new prior) is hard to estimate. At birth, human new-borns already have rudimentary abilities to detect temporal synchrony or spatial coincidence between visual and auditory signals (Morrongiello et al., 1998; Lewkowicz et al., 2010). These provide the basis for learning the associations between newly seen and heard stimuli. By 12 months of age, infants are able to learn about two new visual objects, that each produces a characteristic sound. This occurs after a training period of less than 10 min if tested immediately (Baumgartner and Oakes, 2011). This is the age by which the infants have perhaps developed sufficient cognitive capacities and knowledge to underpin such rapid audiovisual association learning. It should be noted that while such associative learning is rapidly acquired, it is perhaps forgotten quickly too if follow-up experience is lacking. The formation of a unity assumption (and so, a new prior) that can influence human perception rapidly or over a longer time scale needs to take the human developmental trajectory and brain plasticity into consideration (see Murray et al., 2016).

In one of the most convincing studies using the prior term in a Bayesian model to represent the learning of the mapping between crossmodal signals, Ernst (2007) trained the participants with typically unrelated visual and tactile features; specifically, luminance and stiffness. After a training session of about 1.5–2.5 h of exposure, the luminance and stiffness dimensions became correlated, leading to better performance in the congruent (i.e., the trained pairing) than in the incongruent condition. He called this newly learned mapping between the visual and tactile features a ‘coupling prior,’ which leads to a higher likelihood of integrating the multisensory signals.

According to the Bayesian causal inference model, the *prior of common cause* (*p*_common_) refers to “*how likely two co-occurring signals are to have a common cause vs. two independent causes*” (Körding et al., 2007, p. 3). This is close to the notion of the *unity assumption*. On the other hand, the *coupling prior* that was established in Ernst’s (2007) study refers to the participants’ knowledge of “*mapping uncertainly between the two signals*,” which is closer to the idea of *crossmodal correspondence*. Such differences raise a problem for the coupling prior: when exactly one should consider that there is sufficient evidence for the unity assumption to emerge? What is more, it would appear that the notion of coupling priors makes no assumption as to whether two stimuli belong to the same object or not; instead, all that is entailed is that the stimuli are correlated.

### Interim Summary

The research that has been reviewed in this section highlights the computational approach in modeling the unity assumption according to the Bayesian causal inference model using a prior term (*p*_common_). Ernst (2007) has demonstrated that even a couple of hours of exposure to co-occurring visual and tactile stimuli can lead to a stronger coupling prior between them. Nevertheless, the coupling prior seems no more than a correlation or association between the stimuli, and the concern that whether crossmodal signals that are associated (or congruent) would be integrated as a unified object/event remains (see the section “Crossmodal Correspondences”).

## The Unity Effect: Outstanding Issues

Having reviewed the core literature relevant to assessing the impact of the unity assumption on multisensory integration, all that remains is to discuss a number of outstanding issues in the area that have yet to be resolved.

### The Unity Effect – All a Matter of Definition?

According to Welch and Warren’s (1980) original definition, the ‘unity assumption’ was used as a term to refer to those situations in which an observer believed that the various unisensory stimuli with which they had been presented belonged to one and the same object or event. Hence, research on crossmodal correspondences would not necessarily be relevant. This is because while people do indeed believe that different sensory cues can be mapped between two continuous unisensory dimensions or categories, they do not necessarily think that the stimuli belong to one and the same object or event. To make the distinction absolutely clear, while most people would choose ‘bouba’ as the appropriate matching for a rounded cloud-like shape and ‘kiki,’ for an angular star-like shape (see Ramachandran and Hubbard, 2001; Bremner et al., 2013; Chen et al., 2016), they do not necessarily believe that those are the names of those shapes.

Similarly, most of the research that has been published to date on the topic of semantic congruency effects is also irrelevant to the debate concerning the unity effect given a strict definition of the phenomenon (e.g., Connolly, 2014). Most researchers studying semantic congruency have chosen to present pairs of stimuli that, while they refer to the same concept (e.g., dog), do not necessarily refer to the same specific multisensory object or event. That is, no one is likely to think that the line drawing of a dog is the source of the barking sound that they hear in a typical semantic congruency experiment.^9^

However, here we would like to argue that this uncertainty doesn’t matter too much when it comes to evaluating the effects of the unity assumption. Considering that the fundamental question is how the unity assumption helps solve the crossmodal binding problem, it is essential to understand the condition in which each factor would work. Therefore, rather than restricting ourselves to a narrow definition, we favor the alternative position. That is, to broaden the definition of the unity assumption to include any factor that may lead to people’s believing that two or more stimuli ‘belong together.’ Then, in future studies, it would be critical to clearly specify the source that leads to the formation of unity assumption in the observer’s mind, and try to quantify the influence of each source.

### Is Speech Special in Terms of the Unity Effect?

As has been mentioned already, some have wanted to suggest that speech is perhaps special. This is because the unity effect can be reliably observed for speech stimuli under the condition that spatial and temporal coincidence can be violated (reviewed in the sections of The Spatial and Temporal Ventriloquism Effect). Nevertheless, at least as far as the unity effect goes, more recent research shows that the unity assumption induced by emotional valence is perhaps also powerful. For example, in Petrini et al.’s (2010) study, participants had to rate the emotional valence of a video showing a drummer or saxophonist playing a musical instrument. Performance in this emotion rating task was worse (i.e., slower reaction time and/or less accurate) when the video was paired with sound produced by the same instrument but with an incongruent emotional valence, even when the video and the sound did not correspond temporally.

Integrating visual and auditory emotion signals, as compared to speech perception, has received far less attention in studies of multisensory perception (e.g., Massaro and Egan, 1996; de Gelder and Vroomen, 2000; Vroomen et al., 2001; Föcker et al., 2011; Maiworm et al., 2012). Nevertheless, both speech and emotion perception are critical in our daily social interactions, and they rely heavily on the integration of information conveyed by face/lips movements in vision and vocal features in audition. Therefore, speech and emotion are likely underpinned by a common mechanism for integrating multisensory information from face and voice (see Massaro and Cohen, 2000; Campanella and Belin, 2007).

### Is the Unity Assumption a Conscious Belief?

A separate issue here concerns the question of whether anything depends on the observer’s conscious awareness about the relationship between the relevant unisensory inputs. For example, the first author in Arnold et al.’s (2005) study took part in the experiment and reported adopting a strategy of imagining that asynchronous pairs of auditory and visual stimuli originated from the same distal event. His performance then demonstrated a perceptual compensation for the travel time difference of light and sound, whereas other naïve participants did not. Presumably, accepting that merely the observer’s conscious belief^10^ regarding certain stimuli belonging together were able to change the degree of multisensory integration would take us into the issue of cognitive penetrability (e.g., Macpherson, 2012), and ‘the new look’ movement (e.g., Francis, 2012). It would seem, though, that despite the fact that researchers have been talking about the influence of the unity assumption for decades now, a clear consensus has still not been reached as to whether the observer is required to be consciously aware of the mapping between the component sensory stimuli or not. The early work of Welch and Warren (1980) certainly made it seem like this was the case. However, nowadays, this condition seems to have fallen by the wayside (see the section “Factors Leading to the Unity Assumption”).

In any case, the unity assumption provides a clear example of the top–down modulation of human multisensory perception. This mechanism can fit into one of the most intriguing modern views of human perception, known as *predictive coding*. This view suggests that human brain is proactive in predicting the state of the outside world and so the incoming sensory signals; the received sensory signals, in turn, provide sensory evidence to verify the predictions (e.g., Friston, 2005; Bar, 2007; Friston et al., 2015; Kok and de Lange, 2015). The unity assumption, either generated by one’s beliefs, prior experience, or the features of the stimuli, modulates the incoming multisensory signals to be either integrated (i.e., giving rise to the unity effect) or not, and therefore can be considered as a sort of prediction. Theories of predictive coding, nevertheless, do not necessarily consider the top–down modulations at the conscious level (see Macpherson, 2017). The predictive coding view would also provide a theoretical basis to examine unity assumption in modern neuroscience approach (e.g., Gau and Noppeney, 2016) and Bayesian modeling approach (e.g., Odegaard and Shams, 2016) in future research.

### Does the Unity Effect Require Experience?

Welch and Warren (1980; see **Figure 1**) included one box in their model for experimental instructions. Nevertheless, it has been shown to be insufficient to induce a unity effect based simply on instruction from the experimenter (Warren et al., 1981). This raises the question of how the unity assumption can be formed and updated by experiencing the co-occurrence of the relevant unisensory stimuli, in addition to through what is said by the experimenter to the participants?

In Nahorna et al.’s (2012, 2015) studies, the participants’ tendency to integrate audiovisual speech information can be induced (or reversed) by the context presented a few hundreds of ms before the target stimuli. Additionally, Wozny and Shams (2011) have demonstrated that the experience of unity for arbitrarily paired visual and auditory stimuli can modulate subsequent perception on a trial-by-trial basis. Note here that the speed of forming the unity assumption (or prior of common cause) may not be comparable for different combinations of sensory modalities because cross-talk between different senses are unequal (it could be hypothesized, say, that audiovisual interplay should be most extensive). Taken together, the unity assumption for two newly paired crossmodal stimuli can form rapidly in terms of experience; however, the remaining question is how strong it is and whether it builds up over successive exposures.

### Does attention Give Rise to a “Unity Effect”?

Attention has been taken to be a critical cognitive factor in modulating multisensory integration (see **Figure 1**; see Talsma et al., 2010, for a review). Previous research has demonstrated that the effect of multisensory integration is more pronounced when the participants attend to stimuli in both sensory modalities rather than focusing on just the stimulus in one modality (e.g., Talsma et al., 2007; Mozolic et al., 2008; Fairhall and Macaluso, 2009). At first glance, such evidence suggests that attending to multiple sensory modalities should enhance the multisensory integration (i.e., leading to a unity effect).

A recent study using Bayesian models to quantify the prior of common cause (*p*_common_) suggests that attention does not seem to increase the tendency of multisensory integration. Odegaard et al. (2016) examined the influence of attention on the spatial ventriloquism effect. The result demonstrates that the reliability of the visual or auditory signal was higher when the participants attended to that modality than when they divided their attention between both modalities; however, the prior of common cause did not significantly change in the focus vs. divided attention condition. Other studies have manipulated the participants’ attentional resources using a single vs. dual tasking manipulation, and the results demonstrated the participants’ performance remained statistically optimal in both conditions (Helbig and Ernst, 2008; Vercillo and Gori, 2015; Wahn and König, 2016). Taken together, these results therefore suggest that the allocation of attention across sensory modalities or tasks does not influence the tendency toward binding multisensory signals. That is, attention and the unity assumption seem to be two different mechanisms in multisensory integration.

### Has the ‘Unity Assumption’ Fallen Out of Fashion?

Given these various concerns, it would seem legitimate at this point to consider whether we wouldn’t simply be better off dispensing with the very notion of the unity assumption. What exactly, one might ask, would be lost were we to scrap the term, and instead simply replace it with the notion of the prior term from the Bayesian approach (e.g., Ernst, 2007; Körding et al., 2007; Beierholm et al., 2009; Rohe and Noppeney, 2015a,b)? Indeed, this review reveals that the unity assumption can originate from heterogeneous causes. In addition, it remains unclear whether the unity assumption relates to an observer’s conscious belief, or just an implicit sense that the multisensory sensory inputs belong together. Finally, to date, the behavioral data fail to tell a clear story about (or to predict) precisely which conditions, or stimuli, will give rise to a unity effect, and which will not.

**FIGURE 7:**
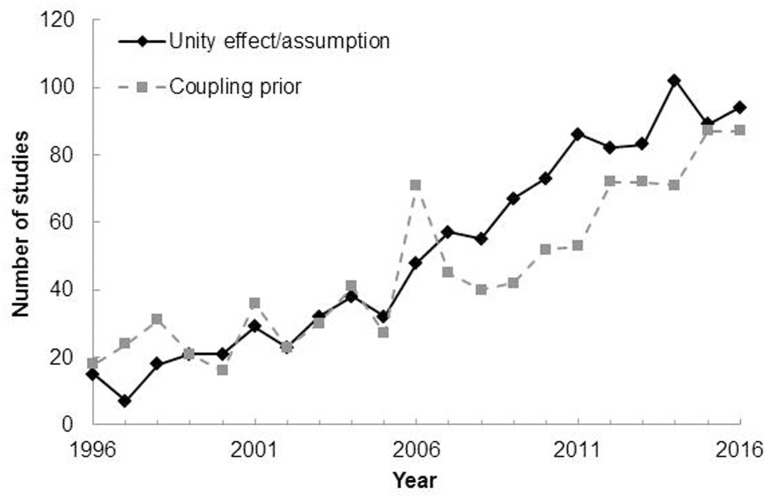
**Number of published articles including the word of “unity effect” (or “unity assumption”) and “coupling prior” in the title, abstract, or in the text of papers listed in Google Scholar in the past 20 years.** The use of both terms has been rising slowly but surely in recent years, thus arguing against those claim that the notion of the unity effect/assumption has fallen out of fashion in recent years.

Given all of the above, some might say that the research field has already implicitly eliminated all further discussion of the ‘unity assumption’. Nevertheless, a Google Scholar search on the term unity effect/unity assumption in the title, abstract, or in the text of papers reveals an ever increasing number of hits along with the term ‘coupling prior’ (see **Figure 7**).

## Conclusion

Thus, in conclusion, in this review we have demonstrated that the unity assumption influences multisensory integration across a range of stimulus pairs from multiple sensory modalities. We have put forward the view that, in addition to the experimenter’s instructions, the literature on crossmodal correspondences and semantic congruency, can all potentially be subsumed with the debate on the unity assumption. The evidence primarily comes from several experimental paradigms including the spatial and temporal ventriloquism effect, and the McGurk effect. Hence, it is clear that the unity assumption genuinely modulates human behavioral performance, especially in audiovisual speech perception. Finally, we have reviewed the evidence from those studies that have used Bayesian models to simulate human multisensory integration, in which the prior of common cause, a variable that represents the probability of two signals going together, can be linked to the unity assumption discussed within current cognitive frameworks. As such, we would argue that the prior of common cause provides a novel means of quantifying the unity effect in cognitive models in future research.

## Author Contributions

Y-CC and CS both contributed equally to the writing of this review paper.

## Conflict of Interest Statement

The authors declare that the research was conducted in the absence of any commercial or financial relationships that could be construed as a potential conflict of interest.

## Footnotes

1 Instead of simply using the term “object,” which may implicitly refer to a concrete material thing (see Kubovy and van Valkenburg, 2001), the term “event” is used here in order to emphasize the temporal property that is typically associated with multisensory stimulation and which can be defined by the onset and offset of a stimulus giving rise to a dynamic change, such as someone opening his/her mouth to produce a word.

2 The use of the term ‘assumption’ or ‘belief’ might lead one to suggest that this modulatory factor relies on the observer being consciously aware of the connection, or relation, between the component unisensory signals. Such a view is encouraged by the fact that many of the examples cited by Welch and Warren (1980) in their early review involved those situations in which the participants were explicitly provided with the unity assumption by the experimenter. However, at the outset it would seem more parsimonious to remain agnostic on this issue (see Deroy, 2013). That is, to assume that the unity assumption need not be represented consciously, although, of course, sometimes it might be (e.g., Klapetek et al., 2012; Faivre et al., 2014).

3 Using human speech stimuli and naturalistic events like balls bouncing off a surface, Vatakis and Spence (2007, 2008) framed their results in terms of ‘the unity effect.’ One could, though, perhaps also describe their study in terms of semantic congruency effects. The distinction (or perhaps, better said, convention) here is that when the same object or environmental event is involved then the unity effect is evoked, whereas when the same category but not necessarily the specific exemplar object or event is presented, then semantic congruency is more commonly used.

4 Here, it is perhaps worth noting that the TOJ task actively encourages participants to try and keep the component unisensory stimuli segregated. By contrast, the simultaneity judgment (SJ) task, wherein participants have to judge whether two stimuli were presented at the same time or not can be seen as encouraging the observer to try and bind the stimuli together instead. Hence, any researcher who demonstrates a unity effect in an SJ task would leave him/herself open to the criticism that their results reflected nothing more than a response bias (a bias to assume that the stimuli went together). Here it should be noted that there is an on-going debate in the literature as to whether performance on these two tasks gives rise to compatible results (e.g., van Eijk et al., 2008; García-Pérez and Alcalá-Quintana, 2012; Love et al., 2013).

5 In such cases, the associations between visual and auditory signals are twofold: in addition to the semantic congruency (i.e., the dog image and the barking sound), there is also a causal relationship (i.e., the dog open its mouth to *produce* the barking sound). The pairs of stimuli that are semantically congruent or causally related, nevertheless, may give rise to different perceptual outcomes, especially in the temporal domain. For example, in a ball bouncing event, human participants were *less* sensitive to the temporal asynchrony when the visual collision was presented before the impact sound, as compared to the reversed condition of the sound leading the visual collision (e.g., van Eijk et al., 2008; Schutz and Kubovy, 2009; Mitterer and Jesse, 2010).

6 If the temporal correlation between the visual and auditory stimuli was sufficient to elicit a strong unity effect, the implication would be that the unity effect on spatial ventriloquism reported by Jackson (1953) should be attributed to the rich temporal structure rather than necessarily to the semantic congruency between the pairing of the sound of a steam whistle and the sight of a steaming kettle.

7 On the other hand, though, one might wonder whether certain speech sounds might not give rise to a larger ventriloquism effect than others due simply to the higher temporal correlations (Parise et al., 2012). This factor, nevertheless, was not controlled with the stimuli used by Kanaya and Yokosawa (2011).

8 This feature is consistent with the fact that the window in which audiovisual simultaneity is perceived is typically wider when vision leads than when audition leads (Zampini et al., 2005). However, the width of the temporal windows of the McGurk effect and audiovisual simultaneity seem to be different (van Wassenhove et al., 2007; Soto-Faraco and Alsius, 2009).

9 Note that one other problem that raises its head at this point is that the very definition of a multisensory object is itself problematic (Spence et al., 2014), with some researchers (e.g., Busse et al., 2005; Turatto et al., 2005) seemingly being more than happy to treat otherwise unrelated auditory and visual stimuli as constituting a multisensory object, just as long as they occur at more or less the same place or the same time. We certainly do not wish to endorse such a position. However, a full discussion of this issue, i.e., how to define a multisensory object, if they exist, will take another article to work though.

10 Welch and Warren’s (1980) early paper had a section entitled the “Awareness of the discrepancy” that is relevant here. The evidence reviewed there was taken to support the claim that the multisensory integration of visual and proprioceptive cues, as compared to visual and auditory cues, is somewhat less susceptible to the participants’ awareness of the multisensory discrepancy.
